# Optical Coherence Tomography Angiography in Papilledema Compared With Pseudopapilledema

**DOI:** 10.1167/iovs.18-25453

**Published:** 2019-01

**Authors:** Masoud Aghsaei Fard, Alireza Sahraiyan, Jalil Jalili, Marjane Hejazi, Yanin Suwan, Robert Ritch, Prem S. Subramanian

**Affiliations:** 1 Farabi Eye Hospital, Tehran University of Medical Science, Tehran, Iran; 2 Department of Medical Physics and Biomedical Engineering, Research Center for Molecular and Cellular in Imaging, School of Medicine, Tehran University of Medical Science, Tehran, Iran; 3 Department of Ophthalmology, Ramathibodi Hospital, Mahidol University, Bangkok, Thailand; 4 Einhorn Clinical Research Center, New York Eye and Ear Infirmary of Mount Sinai, New York, New York, United States; 5 Department of Ophthalmology, University of Colorado, School of Medicine, Aurora, Colorado, United States

**Keywords:** papilledema, pseudopapilledema, vessel density

## Abstract

**Purpose:**

The purpose of this study is to evaluate differences in optical coherence tomography angiography (OCT-A) findings between patients with papilledema and pseudopapilledema.

**Methods:**

In this prospective, comparative study, 41 eyes of 21 subjects with papilledema, 27 eyes of 15 subjects with pseudopapilledema, and 44 eyes of 44 healthy normal subjects were included and were imaged using OCT-A. In addition to peripapillary total vasculature maps obtained with commercial vessel density mapping, major vessel removal using customized image analysis software was also used to measure whole image capillary density and peripapillary capillary density (PCD). Peripapiilary retinal nerve fiber layer (RNFL) and macular ganglion cell complex (GCC) were recorded.

**Results:**

Average RNFL thicknesses were greater in papilledema eyes than in pseudopapilledema and control subjects. GCC thickness was not different among three groups. Peripapillary vasculature values were significantly lower in papilledema (58.5 ± 6.1%) and pseudopapilledema (58.9 ± 4.7%) eyes compared with healthy eyes (63.2 ± 3.1%) using commercial machine software, without a difference between papilledema and pseudopapilledema eyes. However, using our customized software, peripapillary “capillary” density of papilledema eyes was 29.8 ± 9.4%, which was not significantly different from healthy subjects (31.8 ± 7.4%; *P* = 0.94). Pseudopapilledema eyes with peripapillary density of 25.5 ± 8.3% had significantly lower capillary values compared with control eyes (*P* = 0.01). There was a significantly lower whole image and nasal sector peripapillary capillary density of inner retina in pseudopapilledema eyes than papilledema eyes (*P* = 0.03 and *P* = 0.02, respectively).

**Conclusions:**

Whole image and nasal peripapillary sector capillary densities using OCT-A had diagnostic accuracy for differentiating true and pseudo-disc swelling.

Papilledema is swelling of the optic nerve head resulting from axonal stasis due to elevated ICP. The differentiation of pseudopapilledema caused by congenital optic disc elevation (crowded optic disc) or optic disc drusen from papilledema is of critical importance, because papilledema may indicate a life-threatening condition such as obstructive hydrocephalus, meningitis, or brain tumor.^1^ Distinguishing papilledema from pseudopapilledema may be difficult despite ophthalmoscopic features of the latter such as dome-shape disc elevation, clear peripapillary nerve fiber layer, and anomalous peripapillary vessels.^1^,^2^ Therefore, ancillary testing has been suggested to aid in differentiating papilledema and pseudopapilledema.

Fluorescein angiography of eyes with pseudopapilledema shows early and late nodular staining of the optic nerve head, in contrast to leakage that occurs with papilledema.^3^–^5^ Spectral-domain optical coherence tomography (OCT) in papilledema also demonstrates thickening of the peripapillary retinal nerve fiber layer (RNFL), as well as thinning of the macular ganglion cell complex (GCC), which might be useful to distinguish from pseudopapilledema.^6^–^11^ We previously showed that peripapillary total retinal volume is a useful measure to differentiate congenitally elevated optic discs from mild papilledema.^12^

OCT angiography (OCT-A) permits a quantitative, albeit static assessment of macular and peripapillary vascular structures.^13^,^14^ Previously, we reported reduction of peripapillary vessel density in ischemic optic neuropathy and different types of glaucoma using OCT-A.^15^–^18^ Because the true axonal swelling in papilledema may compress adjacent vascular structures (evident ophthalmoscopically in severe cases as venular congestion), we postulated that OCT-A might reveal differences between eyes with true disc swelling and pseudopapilledema, despite a similar ophthalmoscopic appearance. In addition, we designed custom image analysis software that removes the contribution of large peripapillary vessels,^15^–^18^ which might be distorted and obscured in disc swelling, to improve our ability to detect true differences in capillary structures in the two conditions. Thus, our goal was to identify possible OCT-A vessel density differences between subjects with pseudopapilledema and papilledema.

## Materials and Methods

### Subjects

Patients with optic disc elevation due to pseudopapilledema or papilledema secondary to idiopathic intracranial hypertension seen between February 2016 and December 2017 were enrolled in this prospective, comparative study. The study was approved by the Ethics Committee of Tehran University of Medical Science, and all investigations adhered to the tenets of the Declaration of Helsinki. Each subject provided written consent to participate in the study after being informed of the study protocol and requirement.

Diagnosis of papilledema and pseudopapilledema was established by one of authors (MAF) based on evaluation of the patient's history and review of diagnostic testing such as neuroimaging and lumbar puncture, if available. The papilledema group consisted of patients with idiopathic intracranial hypertension (before starting medication) with lumbar punctures showing ICP >250 mm H_2_O and with normal magnetic resonance imaging (MRI) and magnetic resonance venography. Patients with grade 4 and 5 of Frisén staging^19^ papilledema and chronic papilledema as defined by swelling with pallor and/or macular exudate, and/or patients with a >6-month duration of diagnosed papilledema were excluded. Inclusion criteria for pseudopapilledema were congenital optic disc elevation or a crowded optic disc with or without optic nerve head drusen visible by ophthalmoscopy or B-scan. Those subjects without optic disc drusen must have had stable optic nerve appearance during at least a 6-month follow-up or ICP of less than 250 cm H_2_O.^5^,^12^

The control group, collected during a parallel study,^18^ comprised age-matched subjects with a best-corrected visual acuity of ≥20/30, normal optic disc appearance, and IOP ≤21 mm Hg.

In all three groups, patients with refractive errors ≥+6.00 or ≤−6.00 D or more than ±3.00 D astigmatism, a history of ocular surgery (except for uncomplicated cataract surgery), or a glaucomatous or neurologic disease were also excluded.

### OCT Measurements

As in our prior study,^18^ peripapillary RNFL and macular GCC images were acquired in all subjects following pupillary dilation, using the Optovue OCT (software version 2016.1.0.90, AngioVue; Optovue, Inc., Fremont, CA, USA) with signal strength index of more than 48.

Standard 360°, 3.4-mm-diameter circular scans were used to record average RNFL and sextant thickness values. Macular GCC thickness was reported as superior, inferior, and total GCC thickness.

## OCT-A

**Figure 1:**
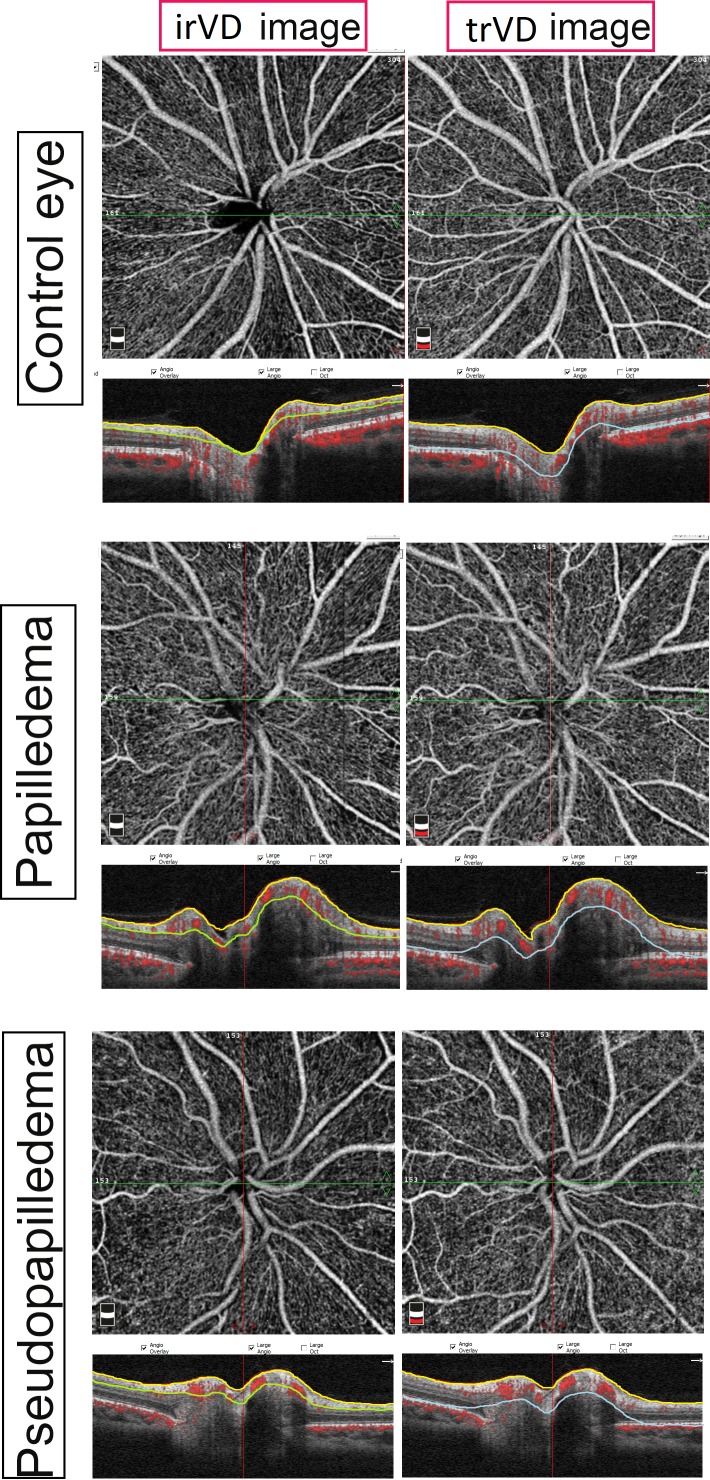
OCT-A irVD and trVD images with accompanying B-scan images of control, papilledema, and pseudopapilledema eyes. irVD and trVD images were acquired between internal limiting membrane (yellow line) and nerve fiber layer (green line), and internal limiting membrane and retinal pigment epithelium (blue line), respectively, which were accurately segmented in papilledema and pseudopapilledema eyes.

**Figure 2:**
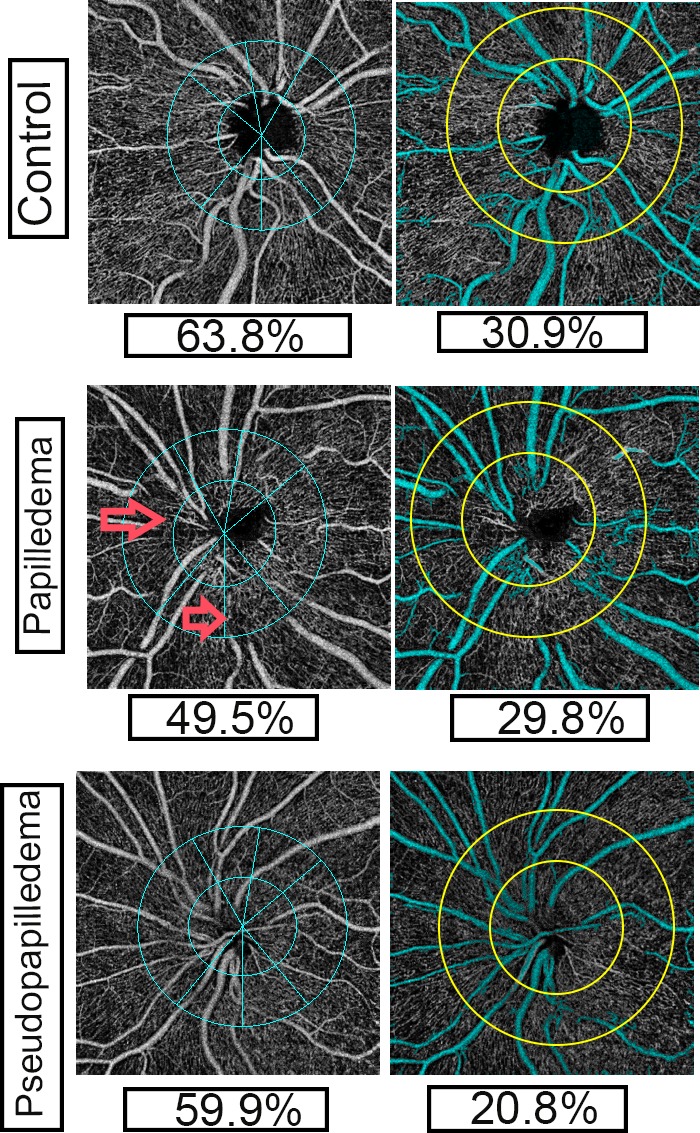
OCT-A of peripapillary total vasculature (left column) and capillaries (right column) of inner retinal images in control, papilledema, and pseudopapilledema eyes. Left column: Commercial OCT-A (Optovue) sectors of peripapillary vasculature with peripapillary total vasculature density shown. Right column: OCT-A images with two concentric circles with 3.45- and 1.95-mm-diameter with customized software; major vessel (in cyan) removed using customized software. Peripapillary capillary densities were also shown. Capillary density in pseudopapilledema eyes is lower than papilledema and control eyes. Large vessel distortions and obscurations are seen in OCT-A images of papilledema (red arrows) compared with pseudopapilledema eyes.

Please see our prior studies^15^–^18^ for a detailed description of OCT-A image acquisition. In brief, a 4.5- × 4.5-mm rectangle scan centered on the optic disc was used to record OCT-A images (with signal strength index of more than 48) using the AngioVue split spectrum amplitude-decorrelation angiography (SSADA) algorithm. Both large vessel and capillary densities from the internal limiting membrane (ILM) to the RNFL posterior boundary are imaged by the standard AngioVue software, and this measurement has been termed the retinal peripapillary capillary density (RPC) image. In this article, we used inner retinal vessel density (irVD) term for this type of image. Total peripapillary vascular (capillary and large vessels) density from ILM to RPE also was recorded and termed ONH image, and we used total retinal thickness vessel density (trVD) for this image. Total peripapillary (between commercially placed concentric circles) and its six sector (superotemporal, superonasal, temporal, nasal, inferotemporal, and inferonasal) vascular density values in irVD and trVD were reported (Figs. 1, 2). We then used customized software^15^–^18^ to determine “actual” capillary density in the same layers. OCT-A images (both irVD and trVD) were analyzed using a custom MATLAB program (The Mathworks, Inc., Natick, MA, USA) to calculate whole image capillary and peripapillary capillary densities (PCD) as we described before.^16^ In summary, after placing two concentric circles with 3.45- and 1.95-mm diameters and removing large vessel signals, capillary density was calculated in both irVD and trVD images using a thresholding technique. Whole image capillary density (including the disc area) and PCD (between the two rings, excluding the disc area) were measured.^18^ The PCD ring was also divided into four sectors (superior, inferior, nasal, and temporal), and their values were reported (Fig. 2).

### Statistical Analysis

Numerical data distribution was analyzed for normality using the Shapiro-Wilk test. Mean and SD were calculated, and categorical variables were compared using the χ^2^ test. Linear mixed modeling was used for the comparison between groups, after accounting for intereye correlation, and Bonferroni correction was performed to adjust for multiple comparisons between groups within each analysis. Areas under the receiver operating characteristic curve (AUCs) was used to determine diagnostic accuracy of vessel density values for differentiation of papilledema and pseudopapilledema.

## Results

Forty-one eyes of 21 patients with papilledema (16 eyes with grade 1 or 2 papilledema and 25 eyes with grade 3 edema), 27 eyes of 15 subjects with pseudopapilledema (10 eyes with optic disc drusen, 17 eyes with optic disc elevation without drusen), and 44 eyes of 44 healthy normal subjects were included in this study after excluding 2 control, 1 pseudopapilledema, and 6 papilledema eyes because of poor signal quality and/or eye movement. Five subjects (10 eyes) with grade 1 or 2 papilledema had been analyzed in a prior study as well.^18^ Table 1 summarizes the demographic information and structural biometric measurements.

**Table 1:** Demographic and Ocular Characteristics of Healthy, Pseudopapilledema, and Papilledema Patients

	**Papilledema (*****n*** **= 41)**	**Pseudopapilledema (*****n*** **= 27)**	**Control (*****n*** **= 44)**	**Papilledema Versus Control**	**Pseudopapilledema Versus Control**	**Papilledema Versus Pseudopapilledema**
Age, y	36.6 ± 12.5	32.2 ± 14.6	39.4 ± 9.8	0.77	0.43	>0.99
Sex, female:male	39:2	17:10	28:16	0.001	0.57	0.001
Visual acuity, logMAR	0.10 ± 0.12	0.07 ± 0.11	0.08 ± 0.14	0.77	0.95	0.58
Average RNFL, μm	150.1 ± 27.9	107.3 ± 16.1	101.7 ± 7.0	<0.001	>0.99	<0.001
Superior RNFL, μm	179.4 ± 44.5	122.8 ± 29.2	122.5 ± 7.9	<0.001	>0.99	<0.001
Nasal RNFL, μm	126.7 ± 41.5	81.3 ± 15.8	83.3 ± 9.3	<0.001	>0.99	<0.001
Inferior RNFL, μm	193.7 ± 35.4	142.8 ± 30.7	125.8 ± 9.8	<0.001	0.16	<0.001
Temporal RNFL, μm	100.4 ± 30.3	82.6 ± 14.5	75.3 ± 8.6	<0.001	0.91	0.002
Total GCC, μm	93.5 ± 11.5	91.3 ± 23.7	96.5 ± 5.5	>0.99	0.68	>0.99
Superior GCC, μm	92.1 ± 11.3	90.6 ± 23.9	96.1 ± 6.1	0.82	0.57	>0.99
Inferior GCC, μm	95.2 ± 12.1	91.9 ± 23.3	96.9 ± 5.3	>0.99	0.80	>0.99

P values are based on linear mixed model.

Average and all sectoral RNFL thicknesses were greater in papilledema eyes than pseudopapilledema and control subjects. RNFL thickness was not statistically different between pseudopapilledema and control eyes. Total GCC thickness was also not different among three groups.

Linear mixed model analysis showed that the whole peripapillary and all sector vessel density values using commercial Optovue software in both irVD and trVD images were significantly lower in papilledema eyes than control eyes (Table 2). There was also a lower vessel density in whole peripapillary and nasal sectors of both trVD and irDV images in pseudopapilledema eyes versus control eyes in addition to lower values of superonasal and temporal sectors of irVD images. All vessel density values were not statistically different between papilledema and pseudopapilledema eyes (Table 2).

**Table 2:** OCT-A Peripapillary Vessel Densities Using Commercial (C) Software in Healthy, Pseudopapilledema, and Papilledema Patients

**Vessel Density in Peripapillary Area (C)**	**Papilledema**	**Pseudopapilledema**	**Control**	**Papilledema Versus Control**	**Pseudopailledema Versus Control**	**Papilledema Versus Pseudopapilledema**
Total retinal VD images						
Whole peripapillary	56.4 **±** 5.9	58.1 **±** 4.3	61.4 **±** 2.3	<0.001	0.01	0.58
Nasal sector	54.9 **±** 6.5	56.9 **±** 5.4	60.7 **±** 2.6	<0.001	0.01	0.53
Inferonasal sector	56.0 **±** 7.6	59.7 **±** 5.0	63.4 **±** 5.1	<0.001	0.06	0.06
Inferotemporal sector	58.2 **±** 7.7	62.4 **±** 4.7	63.8 **±** 3.8	<0.001	>0.99	0.03
Superotemporal sector	58.2 **±** 7.7	60.4 **±** 6.1	62.7 **±** 3.4	0.006	0.39	0.66
Superonasal sector	53.2 **±** 7.6	53.6 **±** 8.8	61.5 **±** 4.1	<0.001	<0.001	>0.99
Temporal sector	57.9 **±** 6.1	58.9 **±** 4.6	59.0 **±** 4.1	>0.99	>0.99	>0.99
Inner retinal VD images						
Whole peripapillary	58.5 **±** 6.1	58.9 **±** 4.7	63.2 **±** 3.1	<0.001	0.002	>0.99
Nasal sector	56.8 **±** 6.8	57.7 **±** 5.6	61.5 **±** 3.1	0.001	0.01	>0.99
Inferonasal sector	59.9 **±** 7.9	61.3 **±** 6.2	65.1 **±** 5.3	0.003	0.07	>0.99
Inferotemporal sector	61.3 **±** 8.4	65.4 **±** 5.1	66.8 **±** 4.4	0.001	>0.99	0.03
Superotemporal sector	60.5 **±** 7.4	62.3 **±** 6.3	65.4 **±** 3.8	0.001	0.12	0.75
Superonasal sector	55.2 **±** 8.3	54.4 **±** 9.0	62.6 **±** 4.7	<0.001	<0.001	>0.99
Temporal sector	59.2 **±** 5.9	58.6 **±** 4.7	62.2 **±** 4.8	0.04	0.02	>0.99

P values are based on linear mixed model. VD, vessel density.

In contrast, eyes with papilledema did not differ significantly from control eyes in whole image capillary density, nor in whole image and all four sector values of both trVD and irVD images using customized software (Table 3). Mean whole image “capillary” density of trVD and irVD images in papilledema eyes was 31.3 ± 9.4% and 28.1 ± 8.2% compared with 32.6 ± 8.1% and 28.8 ± 6.9% in control eyes, respectively (*P* > 0.99 and *P* > 0.94, respectively).

**Table 3:** OCT-A Capillary Densities Using Customized Matlab (M) Software in Healthy, Pseudopapilledema, and Papilledema Patients

**Customized “Capillary” Density (M)**	**Papilledema**	**Pseudopapilledema**	**Control**	**Papilledema Versus Control**	**Pseudopailledema Versus Control**	**Papilledema Versus Pseudopapilledema**
Total retinal VD images						
Whole image	31.3 **±** 9.4	28.3 **±** 12.5	32.6 **±** 8.1	>0.99	0.25	0.69
Whole PCD	33.5 **±** 10.2	29.8 **±** 12.3	34.6 **±** 8.3	>0.99	0.18	0.47
Inferior PCD	31.9 **±** 11.8	28.8 **±** 11.9	33.3 **±** 8.5	>0.99	0.30	0.75
Nasal PCD	33.4 **±** 11.2	28.3 **±** 13.0	32.6 **±** 9.3	>0.99	0.37	0.25
Temporal PCD	37.4 **±** 10.4	34.3 **±** 13.4	37.6 **±** 9.6	>0.99	0.66	0.81
Superior PCD	29.7 **±** 10.4	27.9 **±** 12.7	34.1 **±** 7.8	0.19	0.05	>0.99
Inner retinal VD images						
Whole image	28.1 **±** 8.2	23.2 **±** 6.8	28.8 **±** 6.9	0.94	0.01	0.03
Whole PCD	29.8 **±** 9.4	25.5 **±** 8.3	31.8 **±** 7.4	0.56	0.01	0.14
Inferior PCD	27.8 **±** 12.6	25.4 **±** 9.2	31.1 **±** 7.8	0.35	0.08	0.74
Nasal PCD	28.4 **±** 10.1	21.8 **±** 8.4	28.5 **±** 8.2	>0.99	0.01	0.02
Temporal PCD	34.5 **±** 11.9	30.6 **±** 9.8	36.5 **±** 9.8	0.73	0.09	0.44
Superior PCD	25.5 **±** 9.9	23.1 **±** 8.5	30.5 **±** 7.1	0.02	0.004	0.78

P values are based on linear mixed model. PCD, peripapillary capillary density; VD, vessel density.

There were no significant differences in whole image, whole PCD, and its sectors of trVD images between pseudopapilledema and control eyes using our software. However, capillary densities of whole image, whole PCD, and its nasal and superior sectors of irVD images in pseudopapilledema eyes were 23.2 ± 6.8%, 25.5 ± 8.3%, 21.8 ± 8.4%, and 23.1 ± 8.5% for pseudopapilledema eyes compared with healthy eyes values of 28.8 ± 6.9%, 31.8 ± 7.4%, 28.5 ± 8.2%, 30.5 ± 7.1%, respectively. All four values were significantly lower in pseudopapilledema than in control eyes (*P* = 0.01, *P* = 0.01, *P* = 0.01, and *P* = 0.004, respectively; Table 3).

Comparing papilledema and pseudopapilledema eyes, capillary densities showed significantly lower whole image and nasal PCD of irVD images of pseudopapilledema eyes; whole image and nasal PCD of papilledema eyes were 28.1 ± 8.2% and 28.4 ± 10.1% versus 23.2 ± 6.8% and 21.8 ± 8.4% in pseudopapilledema eyes (*P* = 0.03 and *P* = 0.02, respectively). All other capillary values between papilledema and pseudopapilledema eyes were not statistically different (Fig. 2).

We also divided eyes with papilledema to low grade (grade 1 or 2) and grade 3 and compared them with pseudopapilledema and control eyes (Table 4). Eyes with grade 3 papilledema were not different from pseudopapilledema eyes using our customized software; statistically significant differences were not found in any capillary values (*P* > 0.99 for PCD of irVD and trVD images and nasal PCD). The significant difference was between low-grade papilledema and pseudopapilledema eyes for whole image and PCD customized density of irVD image, which were lower for pseudopapilledema eyes.

**Table 4:** OCT-A Vessel Densities Using Customized Matlab (M) and Commercial (C) Software in Various Grades of Papilledema and Pseudopapilledema and Control Eyes

	**Grade 1,2 PE (*****n*** **= 16)**	**Grade 3 PE (*****n*** **= 25)**	**Pseudo-PE (*****n*** **= 27)**	**Control (*****n*** **= 44)**	**Grade 1,2 PE Versus Control**	**Grade 1,2 PE Versus Pseudo-PE**	**Grade 3 PE Versus Pseudo-PE**	**Grade 1,2 PE Versus Grade 3 PE**
Total retinal VD images								
Whole image capillary (M)	35.7 **±** 9.5	28.5 **±** 8.4	28.3 **±** 12.5	32.6 **±** 8.1	>0.99	0.10	>0.99	0.08
PCD (M)	37.7 **±** 10.5	30.7 **±** 9.2	29.7 **±** 12.3	34.6 **±** 8.3	>0.99	0.08	>0.99	0.11
Peripapillary total vessel (C)	59.3 **±** 4.2	54.5 **±** 6.2	58.1 **±** 4.3	61.4 **±** 2.3	0.74	>0.99	0.04	0.002
Inner retinal VD images								
Whole image capillary (M)	31.7 **±** 8.8	25.9 **±** 7.2	23.3 **±** 6.8	28.8 **±** 6.9	>0.99	0.01	0.80	0.18
PCD (M)	34.2 **±** 9.7	27.0 **±** 8.2	25.5 **±** 8.3	31.8 **±** 7.4	>0.99	0.01	>0.99	0.06
Peripapillary total vessel (C)	61.2 **±** 4.9	56.6 **±** 6.2	58.9 **±** 4.7	63.2 **±** 3.0	>0.99	0.62	0.60	0.01

P values are based on linear mixed model. PE, papilledema; VD, vessel density.

**Figure 3:**
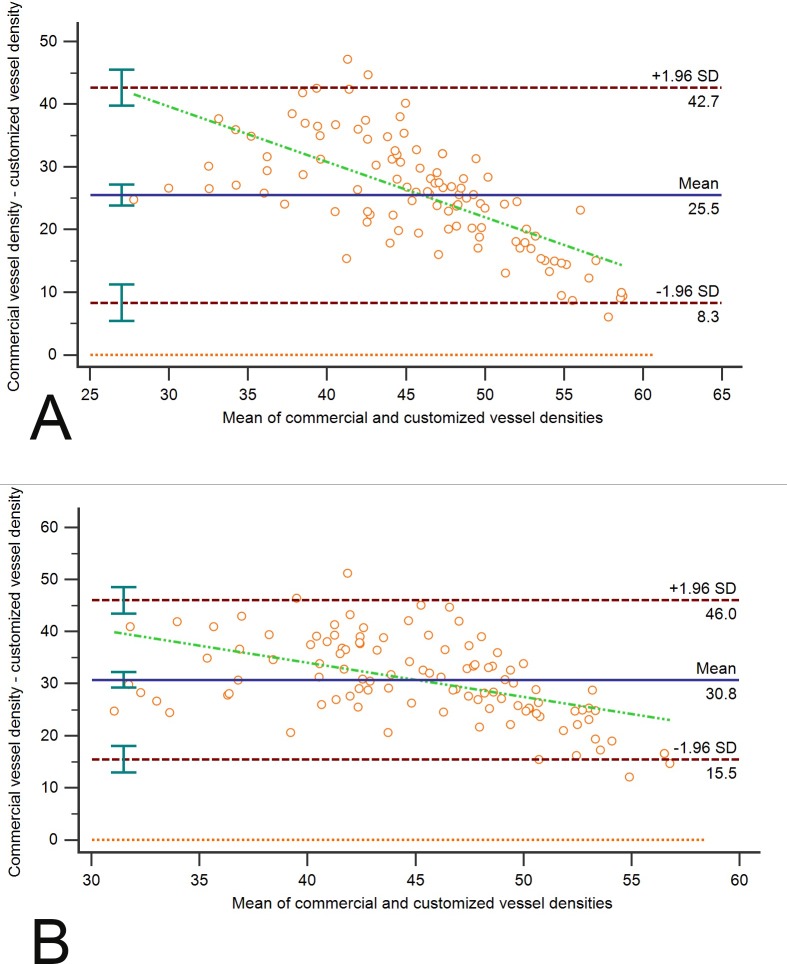
Bland-Altman plot of commercial and customize peripapillary vessel measurements of trVD (A) and irVD (B) images. Solid horizontal line, simple mean difference (25.51% for trVD image, 30.76% for irVD image); dashed horizontal lines, 95% limits of agreement (8.35% to 42.67% for trVD image and 15.5% to 46.02% for irVD image) with 95% CIs. Green dashed line shows correlation between the mean difference and the magnitude of measurements.

Finally, Bland-Altman plots were generated to facilitate comparisons between each method of vessel density measurement. The mean of differences (bias) between customized and commercial measurements was large, and limits of agreement values, representing the range of values in which agreement between methods would lie for approximately 95% of the sample, were large. The bias was 25.5% for peripapillary vessels of trVD image (*P* < 0.001) and 30.7% for peripapillary vessel of irVD image (*P* < 0.001) by two methods. Limits of agreement for trVD image were 8.35% (95% confidence interval [CI], 5.45 to 11.21) to 42.67% (95% CI, 39.81 to 45.57). The irVD images had limits of agreement from 15.5% (95% CI, 12.95 to 18.04) to 46.02% (95% CI, 43.49 to 48.58). We also showed an increase in the bias as the magnitude of the measurement decreases. In trVD and irVD images, the mean differences were correlated to the magnitude of the measurement (*r* = −0.67 and *r* = −0.50, respectively; both *P* < 0.001; Fig. 3). Possible explanation for an increase in the bias as the magnitude of the measurement decreases is that at least the grade 3 papilledema with low vessel densities represented with a fewer large vessel densities, and therefore the larger bias between two methods. To evaluate the effect of image quality on agreement between two measurements, we performed correlation analysis and found association between signal strength index and the differences between two methods of measurements for trVD images (*r* = −0.28, *P* = 0.003). However, such correlation was not found for irVD images data (*r* = −0.17, *P* = 0.07).

Overall, the AUCs for discriminating between papilledema and pseudopapilledema eyes was 0.68 (95% CI, 0.54 to 0.81) for nasal PCD of irVD images and 0.66 (95% CI, 0.53 to 0.80) for whole image of irVD (*P* = 0.01 and *P* = 0.02) with customized software. Nasal PCD of less than 22.6% had 63% sensitivity and 69% specificity for differentiating pseudopapilledema from papilledema. Whole image capillary density of irVD images of less than 22.8% had 74% specificity and 60% sensitivity for differentiating pseudopapilledema from papilledema. AUC for discriminating between papilledema and pseudopapilledema eyes was 0.91 (95% CI, 0.84 to 0.97) for average RNFT thickness (*P* < 0.001).

## Discussion

**Figure 4:**
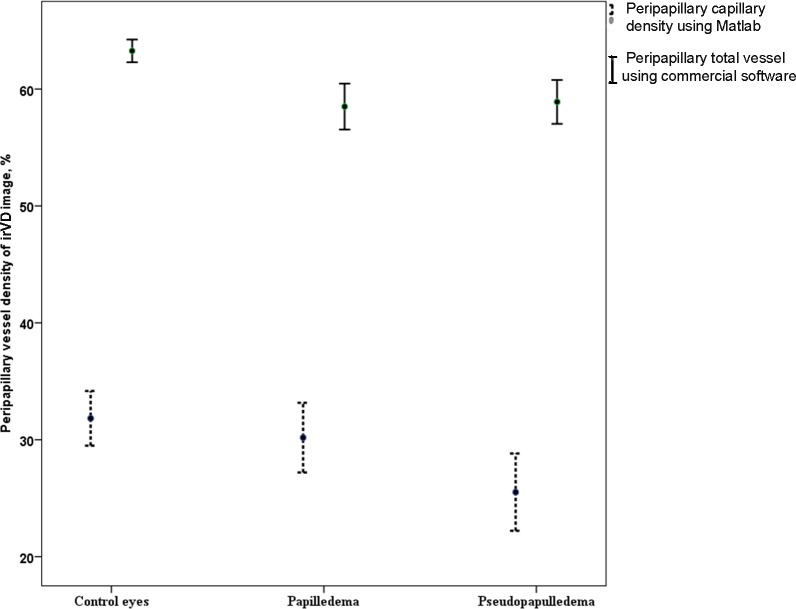
Error bar is representing mean and 95% CI of mean for peripapillary capillary using custom software and total vessels of inner retinal image (irVD image) using commercial software in control, papilledema, and pseudopapilledema eyes.

The quantitative characteristics of small and large peripapillary vessels in the RNFL thickness layer (irVD) and in thicker retinal slab images (trVD) in papilledema, pseudopapilledema, and normal control eyes were evaluated in this study using both commercial and customized OCT-A software. Commercial Optovue software demonstrated a significant reduction in whole peripapillary vessel density values and the vessel density in each sextant, in both irVD and trVD images, in papilledema compared with control eyes. Pseudopapilledema eyes also had lower whole peripapillary and nasal sextant vessels values for the irVD and trVD images, and superonasal and temporal peripapillary vessel densities of irVD images were lower than in control eyes. There was no difference in vessel density between papilledema eyes and pseudopapilledema eyes. We then used a custom program to analyze the capillary density in all eyes. In contrast to the findings with commercial software, the customized software showed that all peripapillary capillary values except the nasal sector of irVD images of papilledema eyes were not different from control eyes. However, in pseudopapilledema eyes, reduced vessel density in irVD images still persisted after excluding the large vessels. In fact, there is a significant decline in whole image and whole peripapillary capillary values and nasal and superior PCD values of pseudopapilledema eyes compared with control eyes (Fig. 4).

Our findings with customized software demonstrate limitations in commercial OCT-A analyses that may limit their utility in evaluation of optic disc pathology. Distortion of large vessels might explain this limitation. Fell D, et al. (*IOVS* 2017;58:ARVO E-Abstract 3305) used custom OCT-A software and reported decreased large vessel density in high-grade papilledema versus low-grade papilledema. Hypothetically, reduced visualization of large vessels on OCT-A might be due to optic disc edema-associated venous stasis within divisions of the central retinal vein. In other words, axoplasmic flow stasis in papilledema and the resulting RNFL swelling secondarily compress the fine, low-pressure venules in that region, resulting in low flow in veins.^20^ Therefore, the apparent reduced density of large vessels on OCT-A might be due to slow flow resulting from venules compression/stasis. However, the reduced arterial flow would be extremely unlikely and does not suggest that it would be the cause of decreased vessel signal on OCT-A. It seems that slow flow has been detected in OCT-A images even in mild papilledema, because our custom software could differentiate grade 1 and 2 papilledema from pseudopapilledema after large vessel removal. By removing the confounding element of large vessel obscuration, our custom analysis software indicates that peripapillary capillary density is not affected in papilledema. Vessel obscuration does not occur in normal eyes or eyes with pseudopapilledema (Fig. 2); the reduced vessel densities in pseudopapilledema eyes detected by both commercial and custom software analyses are therefore from capillary densities. The commercial software fails to differentiate papilledema from pseudopapilledema because it measures reduced large vessel values in the former and reduced capillary values in the latter.

Our customized software data, therefore, demonstrated that whole image capillary density and nasal PCD measured in the irVD image distinguishes between papilledema and pseudopapilledema eyes, and pseudopapilledema eyes had significantly lower PCD values compared with papilledema eyes. Whole image capillary density and nasal PCD of irVD images had AUCs of 0.66 and 0.68 for discriminating between papilledema and pseudopapilledema patients. Nasal PCD of <22.6% had 69% specificity and 63% sensitivity and the whole image capillary of irVD image of <22.8% had 74% specificity and 60% sensitivity for differentiating pseudopapilledema from papilledema. A focal decrease in vessel density within the location of optic nerve head drusen in pseudopapilledema has been shown in one case report,^21^ and overall decreased vessel density in a cohort of 13 patients with optic disc drusen was recently described as well.^22^ Small, elevated, and crowded optic discs in pseudopapilledema might compress peripapillary nerve fiber layer, which then impair retinal blood flow to the peripapillary retina in pseudopapilledema including optic disc drusen.^21^ A similar peripapillary ischemic feature might happen in severe papilledema eyes, accounting for our finding no differences in any capillary values when we compared eyes with grade 3 papilledema with pseudopapilledema eyes. We previously demonstrated significantly decreased PCD in high grade papilledema (grades 3 and 4) compared with grade 1 or 2 papilledema.^18^ Thus, the capillary loss in pseudopapilledema could not be used to distinguish it from higher-grade papilledema. In severe papilledema, swelling of the axons and axoplasmic stasis with resulting vascular compression could lead to nerve ischemia.^1^ In contrast, OCT-A using custom software clearly distinguishes grade 1 and 2 papilledema from pseudopapilledema.

Prior work using fluorescein angiography to differentiate papilledema and pseudopapilledema suggested increased vascularity in papilledema, although 25% of eyes with pseudopapilledema had a similar appearance, and normal control eyes were not studied.^3^ Early hyperfluorescence and late leakage in papilledema may be a sensitive and specific means of distinguishing it from pseudopapilledema, especially in children, because of late disc leakage in papilledema.^5^ Direct comparison between the results of fluorescein angiographic studies and our OCT-A study may not be appropriate, as OCT-A does not measure dynamic values,^23^ and fluorescein angiography cannot visualize the radial peripapillary capillaries that are measured with OCT-A.^24^ Furthermore, OCT-A may be captured rapidly and without need for dye injection; future studies comparing the two angiographic techniques may be helpful to determine their relative abilities to separate these two optic disc abnormalities.

Because the average RNFL thickness in pseudopapilledema eyes was less than papilledema eyes in our study, it seems unlikely that the reduction of PCD measured by OCT-A in pseudopapilledema is due to mechanical impedance of blood flow or signal attenuation secondary to shadowing artifact from disc swelling.^25^ However, the potential effect of edema on signal strength and image quality should not be ignored, as we found a correlation between the image quality score and the disagreement between two methods of measurements. A limitation of our study, and indeed with all studies comparing papilledema with pseudopapilledema, is the use of clinical diagnosis for comparison with imaging tests. Because there is no gold standard imaging tool for detecting pseudopapilledema, a clinical diagnosis that included normal CSF opening pressure or stability of optic disc appearance for at least 6 months was considered the best indicator of the true diagnosis.

In conclusion, we showed that OCT angiography can distinguish papilledema from pseudopapilledema when custom software analysis is used. The vessel density differences, however, are not large enough to enable the clinician to differentiate between papilledema and pseudopapilledema in an individual patient in whom the diagnosis is uncertain. Another potential limitation of our study is a difference in segmentation of irVD and trVD images for controls versus papilledema/pseudopapilledema in the optic nerve head secondary to disc edema that may impact VD for the whole image analyses of custom software. This segmentation problem inside the disc could not be overcome with current Optovue system, which did not allow to adjust the segmentation curves manually and despite all our attempts to perform an accurate segmentation with moving up and down using the segmentation curve, the association of segmentation method with some error was possible.^25^ However, because large vessels inside the disc were removed, most density data in the disc area ignored from whole image density data. Additionally, the comparison between papilledema and pseudopapilledema, both with disc elevation, probably would not be affected by segmentation problem. In addition, we were unable in this study to directly compare the diagnostic ability of OCT-A with our prior work regarding total peripapillary retinal volume, as the OCT device used in this study does not provide such data. Despite the limitations noted above, we find that OCT-A shows promise as a tool to distinguish pseudopapilledema from papilledema. However, further studies are needed to evaluate the effect of different degrees of disc edema on vessel involvements both on the surface and deep optic nerve head structures including choroid. In addition, change in ICP might have effects on peripapillary vascular density, which need prospective studies.
